# Use of an AI-Based Tool (Human Experiences and Reflections Data Connector) to Improve Discovery and Reuse of Archived Qualitative Data: Protocol for an Algorithm Development and Validation Study

**DOI:** 10.2196/82725

**Published:** 2026-04-21

**Authors:** Tammy Leonard, Jim P Stimpson, Miguel Ángel Cano, Wenqi Shi, Ada Abaragu, Song Zhang

**Affiliations:** 1 Department of Health Economics, Systems, and Policy, Peter O'Donnell Jr. School of Public Health, University of Texas Southwestern Medical Center, 5323 Harry Hines Boulevard, Dallas, TX, 75390, United States, 1 2146483155; 2 Harold C. Simmons Comprehensive Cancer Center, University of Texas Southwestern Medical Center, Dallas, TX, United States; 3 Department of Internal Medicine, School of Medicine, University of Texas Southwestern Medical Center, Dallas, TX, United States; 4 Department of Social and Behavioral Sciences, Peter O'Donnell Jr. School of Public Health, University of Texas Southwestern Medical Center, Dallas, TX, United States; 5 Department of Health Data Science and Biostatistics, Peter O'Donnell Jr. School of Public Health, University of Texas Southwestern Medical Center, Dallas, TX, United States

**Keywords:** LLM, large language models, qualitative research, secondary data analysis, information science, transcripts

## Abstract

**Background:**

Despite the growing emphasis on open science and equity in research, qualitative data capturing diverse human experiences and perspectives are rarely reused beyond the original study. Increasingly, data repositories are used to make these data publicly available, but it is unclear whether these data can be effectively identified by researchers interested in secondary data analysis.

**Objective:**

We describe a protocol for identifying and characterizing archived qualitative datasets in leading public repositories, developing an artificial intelligence–based tool to enhance qualitative data reuse, and validating that tool using existing data.

**Methods:**

We will search 4 leading repositories to assess the scope and identifiability of existing publicly available qualitative datasets. We will subsequently build the Human Experiences and Reflections (HEARs) Archive, a directory of deidentified study data that is only accessible indirectly through the use of the HEARs Portal. The HEARs Portal will be supported by large language model–based tools using the retrieval-augmented generation framework. The artificial intelligence tools’ performance will be assessed across 3 domains: relevance of identified studies, validity as evaluated by comparison with human qualitative data analysis, and robustness against the addition of irrelevant information.

**Results:**

A preliminary review of existing data repositories has begun. The anticipated study completion date is December 31, 2026.

**Conclusions:**

The proposed project will provide evidence regarding the existing capacity for identifying and accessing qualitative data through leading repositories. It will also provide evidence on the validity of the HEARs Data Connector for identifying and describing qualitative datasets in ways that can assist researchers interested in secondary analysis. Establishing the validity of the HEARs Data Connector and developing an evidence-based ongoing improvement and monitoring strategy will be essential for establishing trust within the qualitative research community.

## Introduction

### Background

Most scientific research conducted in the health, social, economic, and behavioral sciences, and related fields is aimed at improving human welfare. As public demand for “evidence-based” solutions continues to grow, there is often an implicit emphasis on quantitative evidence. However, qualitative data—data derived from transcribed conversations, open-ended survey questions, or original text from archival materials [1]—are essential for keeping research grounded in real-world experiences and responsive to social and contextual challenges [2]. Qualitative methods can lead to the discovery of determinants that influence behavior, help enhance the scientific rigor of research, facilitate the translation of observational data into interventions, and improve the likelihood that interventions implemented are sustainable and acceptable in the target population [3-7]. Much of the evidence base for programs to improve human welfare is derived from survey or administrative data that are coded numerically and then analyzed using statistical methods. However, data only exist to measure things that some researcher or administrator chose to collect [8]. Qualitative data are the basis of information from which those choices can be best informed. Importantly, qualitative data often return unexpected responses to a question because respondents are not told a priori what response possibilities the researcher has in mind. Qualitative data are necessary to inform accurate interpretation of quantitative results, develop hypotheses and research questions regarding the populations of interest, and improve the relevance of evidence-based solutions—especially those created to serve populations whose perspectives differ from those of the mainstream.

### Importance of Qualitative Data Sharing and Reuse

Qualitative data are costly to collect, and economies of scale for qualitative data collection are less attainable compared to quantitative data. As a result, there are unique economic barriers to increasing the use and impact of qualitative data. Each new instance of interview or focus group data collection requires the same additional researcher time to administer, record, and transcribe the data. In contrast, once quantitative data collection instruments are developed, they can generally be scaled to collect large sample sizes with little additional cost beyond participant recruitment. Despite numerous calls for greater data sharing and reuse and the existence of many large data repositories, secondary analysis of qualitative data is extremely rare. This is regrettably inefficient considering the potential benefits that can come from the reuse of qualitative data and the high cost of primary qualitative data collection.

Increased capacity for sharing and combining qualitative data from multiple studies has been identified as an important goal for improving research reliability and relevance. The Joint Declaration of Data Citation Principles highlights this prioritization within the scientific community by making greater access to data for the purpose of secondary analysis a critical goal [9]. As with quantitative data, data sharing is essential for ensuring that analysis is reproducible. However, this concern is heightened in the case of qualitative data, where analysis can be more heavily influenced by the skill set and perspective of the researcher [10]. Could a different qualitative researcher interpret the same dataset differently? Secondary analysis of qualitative data is also important for facilitating faster dissemination links, whereby new research questions emerge from existing data. Could a researcher from a different disciplinary background analyze the same dataset but extract themes that inform important ongoing work different from the original study? Meta-analysis of qualitative datasets and meta-synthesis of qualitative data findings have shown promise for identifying new research evidence from existing data, but they have largely failed to reach their potential because of the difficulty in identifying and accessing related qualitative studies [11,12]. Finally, qualitative data, when shared, can allow future researchers to identify the early developments of new social challenges that were not even being considered by researchers at the time of data collection [8].

### Challenges to Qualitative Data Sharing and Reuse

Data repositories are increasingly being used to deposit qualitative data for reuse, and some are being created for the sole purpose of facilitating the sharing of qualitative data. However, there remain gaps between the vision (easy sharing and robust reuse of qualitative data) and the reality (sharing is possible, but reuse is infrequent). First, qualitative data are not easy to identify within repositories that also contain quantitative data. Repositories do not generally provide filters that allow searchers to distinguish between quantitative and qualitative data types. Second, across all repository types, qualitative data are often stored behind firewalls to protect the confidentiality of human participants, and institutional review board (IRB) approval is often required just to inspect the data and assess its suitability for a planned research project. Third, guidelines for sharing qualitative data through repositories are varied and often lacking altogether. In a review of 32 English-language multidisciplinary or social science data repositories, only 12 had qualitative data sharing guidelines, and most guidelines remained vague regarding the removal of indirect identifiers [1]. Fourth, when qualitative data can be obtained, it requires additional time inputs to review and assess whether there is sufficient content related to desired themes to warrant use in secondary analysis.

Opportunities related to the better use of qualitative data are being explored. ChatGPT has been shown to perform “reasonably well” in identifying themes within qualitative datasets, but has some noted shortcomings when themes are subtle or require nuanced interpretations [13-15]. However, artificial intelligence (AI) has not been examined as a tool specifically adapted to engage in meta-analysis of qualitative datasets while incorporating core methodological insights of the qualitative field—especially through the development of appropriate metadata to prompt the AI regarding the unique study context and nuance. Researchers are also engaging in greater sharing of qualitative data by building large open-access qualitative datasets to improve monitoring of the evolution of social processes and outcomes. For example, the American Voices Project is building a large, repeated cross-sectional repository of immersive interviews from across the United States.

### The Human Experiences and Reflections Data Connector

The Human Experiences and Reflections (HEARs) Data Connector will fill existing gaps to improve qualitative data sharing and reuse capacity. Importantly, the HEARs Data Connector is intended to complement existing repositories and collaborate with the services they provide. For the purposes of this protocol, we focus on qualitative data collected within research studies, which most often includes transcripts from interviews, focus groups, or ethnographic observations. We also limit our work to English-language text, although we hope to remove this restriction in future studies. The HEARs Data Connector consists of 2 parts: the HEARs Archive and the HEARs Portal.

The HEARs Archive will house deidentified qualitative data, serving as a foundational knowledge base for the HEARs Portal. Unlike traditional data repositories, it will not provide users with direct access to the underlying data. Instead, it will support the Portal’s functionality by enabling data-informed tools and resources. The HEARs Data Connector intends to provide a “middle ground” solution to address the complex challenges related to qualitative data sharing. Deidentified qualitative data can inform summary information accessed by researchers through the HEARs Portal, but direct access to the data itself must be obtained through collaboration with the original investigator or their designee, or by accessing data in an existing public repository. Thus, it allows for data discovery and facilitates reuse, while allowing for heterogeneous, context-specific approaches to ethical data sharing.

The HEARs Portal will be powered by AI and will allow users to perform advanced queries of the HEARs Archive. These queries will generate summaries of key themes within the archive and identify the studies that contributed to each thematic result. To support transparency and reproducibility, the HEARs Portal will display links to the specific studies that contributed to each thematic summary, enabling users to review the source material and assess the analytic pathway. The Portal will provide sufficient detail to support hypothesis generation, guide the selection of qualitative datasets for secondary analysis, and provide information on how to request access to those datasets, such as through links to existing repositories.

The purpose of this protocol is to describe the initial development of the HEARs Data Connector. We will describe the planned foundational research to establish the availability of existing data that can be used within HEARs, as well as the development of the first HEARs demonstration project and its associated performance evaluation.

## Methods

### Study Design

The study protocol is organized into 2 primary phases. Phase 1 encompasses the development and curation of the HEARs Archive, a specialized knowledge base of qualitative research data. This phase involves identifying and acquiring relevant datasets from established repositories, securing investigator permissions, and applying a rigorous preprocessing and deidentification workflow. Phase 2 details the design, implementation, and validation of the HEARs Portal, a web-based tool powered by large language models (LLMs) for querying the HEARs Archive. Specifically, the HEARs Portal allows researchers to query the HEARs Archive, identify related studies from a large sample of qualitative datasets, conduct preliminary descriptive meta-analyses of the datasets in response to queries, and obtain descriptions of the available datasets related to the query (eg, instructions for accessing the data from the investigator or their designee, publications associated with the dataset, study setting, and context). The HEARs Portal will be powered by LLMs with a hybrid retrieval-augmented generation (RAG) framework, whose performance is optimized through synergistic metadata and prompt design. We will assess the performance of the HEARs Portal across 3 domains: relevance of identified studies in the HEARs Archive, validity by comparison with human qualitative data analysis, and robustness against the addition of irrelevant information within the HEARs Archive.

### Patient and Public Involvement

We do not plan for direct patient or public involvement in the designing, conducting, or reporting of this project because its main objective is to develop the HEARs Data Connector, which is primarily a technical task. However, patient and public involvement is anticipated in subsequent projects using and refining the tool for applied use.

#### Phase 1: HEARs Archive

##### Sample Selection

We have identified 4 leading US-based data repositories that are likely to contain qualitative datasets: Open Science Framework, Inter-university Consortium for Political and Social Research, Qualitative Data Repository, and Harvard Dataverse. These repositories are maintained by nonprofit organizations (The Center for Open Science) or academic institutions (University of Michigan, Syracuse University, and Harvard University, respectively) and have been built to promote data sharing among academic researchers. We will first meet with representatives from each repository to understand how best to identify qualitative datasets and use the repositories’ query tools. Next, we will attempt to identify all qualitative datasets available within each repository. From the available studies, we will attempt to identify clusters of topically related studies and select one or more clusters from which to develop and test the HEARs Portal. The goal of identifying clusters is simply to drive efficiency in our initial development of the HEARs Data Connector; thus, we will not use a strict methodology in identifying clusters. We simply seek to find topical areas where there are multiple related studies, and we will prioritize adding studies from these topical areas to the HEARs Archive first to begin development and testing of the portal regarding how it uses information from related studies. We will populate the HEARs Archive with deidentified qualitative datasets, including studies from the identified clusters. We will prioritize populating the HEARs Archive with studies from any repository that are topically related to the cluster that was identified. Before including any study in the HEARs Archive—even studies that are publicly available for download—we will seek permission from the study’s principal investigator and obtain evidence that the data were collected under the supervision of an IRB.

##### Preprocessing and Deidentification

To prepare the raw data for the HEARs Portal, each transcript will undergo a systematic preprocessing and curation workflow to transform it into an indexed, queryable record within the HEARs Archive. First, to ensure participant confidentiality, all transcripts will be deidentified using a Python-based anonymization pipeline and named entity recognition models to automatically detect and redact direct identifiers (eg, names, locations, and so on) and quasi-identifiers. We will test the success of anonymization by manually reviewing a 25% sample of the anonymized data.

##### Repository Structure

Each separate file within the repository will be labeled with a 2-part identifier: study ID and record ID. The 7-digit study ID will be unique to each study. The 4-digit record ID will be unique within each study ID. Metadata will then be created for each 2-part identifier. This will allow metadata to identify both characteristics that apply to an entire study (eg, study settings and study authors) as well as characteristics unique to a particular study record (eg, race or ethnicity of the participant). The repository data will be stored in a secure, encrypted, and password-protected drive in compliance with IRB requirements.

Metadata fields are described in Table 1. Study-level metadata fields include those describing the overall study, the methods used to collect the data, the type and size of study data, and publications related to the study data. Record-level metadata fields describe the record and the characteristics of the participants who provided information for the record. Study-level metadata will be made available so that users of the HEARs Portal can know the full list of studies that are being used. We will implement Schema.org/Dataset via JSON-LD to ensure indexing of metadata can be completed by general search engines like Google Dataset Search, and we will also align our metadata schema with the Data Documentation Initiative Codebook or Lifecycle standard for deep research interoperability. Additionally, we will map our metadata fields (Table 1) by implementing a hybrid ontology mapping that uses the European Language Social Science Thesaurus and MeSH (Medical Subject Headings). Furthermore, the named entity recognition models used in our preprocessing pipeline will be tuned to recognize and align entities with these ontologies during the indexing phase.

**Table 1.** Human Experiences and Reflections (HEARs) Archive metadata fields.

Field category and name		Field level	Example^a^
Study description			
Study ID		Study	7801004
Study principal investigator		Study	John Smith
Study title		Study	Caregiver impact on adherence to discharge instructions
Study purpose or summary		Study	Following an acute illness, patients may be overwhelmed with discharge instructions. This study explores the ability of patients to understand and adhere to instructions upon hospital discharge. A qualitative design of interviews with 63 patients and caregivers was used. Data were collected within 3 weeks of discharge from hospital.
Subject		Study	Medicine, health, and life sciences
Institutional review board of record		Study	University of Texas Southwestern
Study methods			
Study design		Study	Randomized controlled trial
Mode of data collection		Study	Face-to-face interview
Recruitment method		Study	Random sample
Time period		Study	2016-2017
Geographic coverage		Study	Alabama, United States
Study data			
Number of participants in study		Study	126
Number of records		Study	63
Data types		Study	Coded qualitative data
Study publications			
Related publication(s)		Study	Smith, John. 2019. Protocol for Caregiver Impact on Adherence to Discharge Instructions. *Protocol Journal*. 54(2), p111-123
Repository archive		Study	Qualitative data repository
Repository archive identifier		Study	https://doi.org/12.7066/F9MKT6G
Contact for more information		Study	Please refer to instructions at repository.
Record characteristics			
Record identifier		Record	5460
Number of participants in record		Record	2
Data collection method		Record	Interview
Year of data collection		Record	2017
Participant race or ethnic composition			
Non-Hispanic Black		Record	50%
Non-Hispanic White		Record	50%
Asian		Record	0%
Hispanic		Record	0%
American Indian or Alaskan Native		Record	0%
Native Hawaiian or Other Pacific Islander		Record	0%
Participant gender			
Man		Record	50%
Woman		Record	50%
Nonbinary		Record	0%
Participant age (y)			
0‐18		Record	0
19‐65		Record	100
>65		Record	0

a The example provided is not a real study.

### Phase 2: HEARs Portal Design

#### Overview

We will develop the HEARs Portal using a hybrid RAG framework to improve the accuracy and reliability of LLMs for analyzing the HEARs repository [16]. We will leverage the application programming interface–based GPT-series LLMs from the Health Insurance Portability and Accountability Act (HIPAA)-compliant Microsoft Azure OpenAI service as backbone LLMs. The user workflow is explicitly designed as two linked stages: (1) a hybrid retrieval process to identify relevant information and (2) a contextualized generation process to synthesize that information into a coherent response.

The HEARs Portal will identify relevant studies within the repository, summarize key concepts and themes related to the research question contained within these studies, and provide a list of related questions that may be further investigated using the available data in the repository. Users will then have the ability to engage in translation by repeating this process to query competing interpretations and further related questions. With each response, the HEARs Portal will provide a complete list of studies from which the results were obtained, along with summary metadata about the study elements contributing to the response. Importantly, the HEARs Portal will provide a summary of the range of responses to particular questions or thematic analyses alongside the frequency of elements providing support for each response.

#### Stage 1: Hybrid Information Retrieval

We will first create a web interface for querying the AI tool and provide instructions and examples to guide effective use [17]. The instructions will guide users in iteratively using the AI tool in alignment with established methods for meta-analysis of qualitative data steps [11]. When a user submits a research query, the Portal initiates a sophisticated retrieval process designed to locate the most relevant information from the HEARs Archive. To maximize both precision and comprehensiveness, we will use a hybrid retrieval strategy that combines 2 distinct modes of search. First, in explicit search (metadata filtering), users can leverage the rich, structured metadata to perform targeted database-style queries. This allows researchers to narrow the search space to studies or records with specific characteristics (eg, “all interviews with female participants over the age of 65 years from randomized controlled trials”). This acts as a powerful preliminary filter. Second, in implicit search (content-based retrieval)**,** to search the textual content of the transcripts (especially when metadata is incomplete), the Portal will use a combination of state-of-the-art retrieval models to ensure that the results are relevant both lexically and semantically. A sparse retriever, such as a term-frequency model like BM25 [18], will identify documents based on keyword overlap, excelling at matching specific terms. Concurrently, a dense retriever built from text-embedding-3-large embeddings (deployed in our secure Azure environment), with candidate lists fused using reciprocal rank fusion and then reranked using the bge-reranker-large cross-encoder model (Beijing Academy of Artificial Intelligence), will use a semantic similarity model like DRAGON [19] to generate vector embeddings to retrieve passages that are contextually related to the query, even without direct keyword matches. The outputs from both retrievers will be fused and reranked to create an optimized context for the generation stage.

#### Stage 2: Contextualized Generation

In the generation stage, the retrieved text chunks and their associated metadata are fed to an LLM, which synthesizes the information into a coherent, evidence-based narrative. To manage hallucination and ensure traceable attribution, generation will be strictly retrieval-gated with versioned prompt templates that instruct the model to use only the provided evidence and to abstain when evidence is insufficient. Pydantic models will be used to enforce a strict JSON schema on responses, including (1) a theme or summary section, (2) a complete list of contributing study IDs, and (3) chunk-level evidence pointers for each major claim. We will implement automated “no phantom citation” checks that verify every cited study or chunk is present in the retrieved context, and we will treat retrieved transcript text as untrusted input (explicitly disabling instruction-following from the corpus) to reduce prompt-injection risk. The Portal will surface citations as study-level identifiers with repository links and an auditable mapping from each synthesized theme to the supporting studies or records, enabling users to verify provenance and supporting evidence.

While the procedures described are the current most plausible baseline design choices for each component, we recognize that these are inherently empirical decisions; accordingly, we will systematically test reasonable alternatives and select the configuration that performs best on our expert-labeled validation benchmarks.

### Data Analysis

We will validate the HEARs Portal’s performance across several areas: identification of relevant studies, agreement with human analysis of qualitative data, and stability in response to the addition of irrelevant information. First, we will validate that the Portal is able to accurately identify relevant studies and return accurate summaries of the metadata from those studies. To do this, we will assess the accuracy of the retrieval system by comparing its output against a gold-standard evaluation set created by human domain experts. For a diverse set of test queries, we will use standard information retrieval metrics, including precision@k, recall@k, and mean reciprocal rank, to quantify the system’s ability to retrieve relevant documents within the top-ranked results. We will also query the HEARs Portal on subject matter not included in the Archive and test whether the HEARs Portal is able to accurately assess when there is no relevant information available for a given query. Next, we will test for agreement with thematic analysis of qualitative data. We will use peer-reviewed and published studies recording primary analysis of datasets in the HEARs Archive and assess how well the Portal, when examining only a single study, is able to reproduce the results. We will also conduct a meta-analysis of related studies published in the peer-reviewed literature and compare that analysis to results returned from the HEARs Portal when examining data from all studies represented in the meta-analysis. Finally, we will examine the stability of the HEARs Portal by exposing it incrementally to additional studies contained within the full HEARs Archive and repeating the analysis above. We will assess the extent to which responses to queries change when seemingly unrelated, irrelevant studies are added to the corpus of materials examined by the Portal.

### Reproducibility and Sharing

We will develop and maintain Docker containers [20] that capture the complete runtime environment, including all dependencies (Python packages, embedding models, and retrieval frameworks), library versions, and configurations. We will make Docker images available through a public registry (eg, Docker Hub) alongside version-controlled source code. This will enable users and reviewers to verify and reproduce our validation experiments.

Each query of the HEARs portal will generate a persistent ID. The persistent ID will capture the exact query parameters, the specific version of the HEARs Archive queried, and the AI model version used. This will ensure that researchers can cite the specific synthesis provided by the HEARs Portal and thus allow for verification of the analytic pathway.

### Ethical Considerations

This study has been approved by the IRB at the University of Texas Southwestern Medical Center (STU-2024-1045). We have received a waiver of informed consent for our analysis of secondary data. All data will be deidentified and anonymized prior to inclusion in the HEARs archive. We are not enrolling any participants for primary data collection; hence, there is no participant compensation in this study.

### Dissemination Plan

Study results will be disseminated via peer-reviewed studies and presentations at academic conferences. We anticipate at least 2 studies from this work: the first reporting on the availability and ease of accessing qualitative data from existing repositories, and the second reporting on the validation of the HEARs AI tool.

The HEARs Data Connector will become publicly available through a web portal. Users will be required to register prior to using HEARs. We will also work to disseminate our efforts in collaboration with the leadership of other large repositories. Since the HEARs repository will not allow users to access the raw data, we aim to form collaborative relationships with other established repositories so that HEARs can provide incremental benefits by allowing the discovery of qualitative data without redundantly taking on the task of archiving the data for others to access.

Following the initial development and evaluation of the HEARs portal, we will consult with a broad range of stakeholders (eg, repository representatives, researchers, librarians, and funding agency representatives) to discuss our findings and identify next steps for improving HEARs in future projects. We anticipate some of these areas might include the development of strategies to improve the quantity and quality of data in the HEARs archive, investments to improve the sustainability of HEARs, and improvements to metadata and AI tools. Currently, HEARs will be hosted and maintained locally at the University of Texas Southwestern Medical Center; findings from this protocol will inform recommendations for maintaining or adjusting this plan.

## Results

As of February 6, 2026, we have completed work to establish protocols for the identification and processing of studies for inclusion in HEARs. Specifically, we have developed and tested procedures for searching for qualitative studies in existing archives (Open Science Framework, Inter-university Consortium for Political and Social Research, Qualitative Data Repository, and Harvard Dataverse), and we have met with representatives from each of the archives to obtain input on our procedures. We have also developed protocols for receiving studies into the HEARs portal. Additionally, we have developed tools in REDCap (Research Electronic Data Capture, Vanderbilt University) to collect metadata about each study, developed and tested an AI tool to deidentify qualitative data, and piloted all of these processes across 2 existing studies conducted by collaborators of our core research team. We have also begun work to identify qualitative studies available in existing repositories. We anticipate publishing the results of that formative investigation in Fall 2026.

## Discussion

The HEARs Data Connector offers the potential to improve the scientific knowledge creation system by making it easier for existing qualitative data to be reused to inform new study development, in secondary analysis to obtain additional insights, or to provide context in the interpretation of quantitative results when qualitative data were not collected. Because qualitative data are the foundation upon which decisions regarding the scope of quantitative data collection (eg, what survey questions to ask) are made and the interpretation of quantitative data analysis is undertaken, the benefits of being able to reuse existing qualitative data more easily are likely significant. Anticipated findings of the project described in this protocol include the creation of a pilot version of the HEARs Data Connector that can be used to demonstrate its effectiveness in improving capacity for qualitative data reuse.

Obtaining data to populate the HEARs Archive is essential for its long-term success. Our work will illuminate potential gaps in the existing practice of qualitative data sharing as we explore the capacity for including studies from existing repositories into the HEARs Archive. The work is likely to inform new collaborative approaches between researchers and repository managers that can improve the efficiency of secondary analysis of qualitative data. It might also inform ongoing questions regarding the refinement of methods for the secondary analysis of qualitative data [21].

Our project, if successful, will demonstrate an innovative use of AI in qualitative research. Numerous ongoing studies are attempting to use AI to speed up the primary analysis of qualitative data and have identified challenges that the field is working to overcome [22-24]. Our use of AI is more limited in scope and perhaps more suited to the strengths of current-generation AI tools [25], in that we are not attempting to analyze qualitative data with the HEARs Portal, but rather to identify and describe qualitative data resources that can be used in human- or machine-based analysis.

Validation of the initial implementation of the HEARs Data Connector will be essential for establishing efficacy and informing future improvements to the Data Connector. The HEARs Data Connector’s long-term success will largely be determined by the willingness of researchers and repository leaders to adopt the tool [26]. While guidelines for evaluating the ethical and scientifically grounded use of AI for the novel application we propose are not available, our results can be compared to established principles related to AI use in other contexts [27,28]. Establishing the validity of the tool’s output and developing an evidence-based, ongoing improvement and monitoring strategy will be essential for establishing trust within the qualitative research community.

## Abbreviations

AI: artificial intelligence
HEARs: Human Experiences and Reflections
HIPAA: Health Insurance Portability and Accountability Act
IRB: institutional review board
LLM: large language model
MeSH: Medical Subject Headings
RAG: retrieval-augmented generation
REDCap: Research Electronic Data Capture
